# The effect of smoking on chronic inflammation, immune function and blood cell composition

**DOI:** 10.1038/s41598-020-76556-7

**Published:** 2020-11-10

**Authors:** Ingrid Elisia, Vivian Lam, Brandon Cho, Mariah Hay, Michael Yu Li, Michelle Yeung, Luke Bu, William Jia, Nancy Norton, Stephen Lam, Gerald Krystal

**Affiliations:** 1 The Terry Fox Laboratory, British Columbia Cancer Research Centre, Vancouver, BC Canada; 2 Brain Research Centre, University of British Columbia, Vancouver, BC Canada; 3 Department of Integrative Oncology, British Columbia Cancer Research Centre, Vancouver, BC Canada

**Keywords:** Cancer, Immunology, Biomarkers, Risk factors

## Abstract

Smoking is the number one risk factor for cancer mortality but only 15–20% of heavy smokers develop lung cancer. It would, therefore, be of great benefit to identify those at high risk early on so that preventative measures can be initiated. To investigate this, we evaluated the effects of smoking on inflammatory markers, innate and adaptive immune responses to bacterial and viral challenges and blood cell composition. We found that plasma samples from 30 heavy smokers (16 men and 14 women) had significantly higher CRP, fibrinogen, IL-6 and CEA levels than 36 non-smoking controls. Whole blood samples from smokers, incubated for 7 h at 37 °C in the absence of any exogenous stimuli, secreted significantly higher levels of IL-8 and a number of other cytokines/chemokines than non-smokers. When challenged for 7 h with *E. coli,* whole blood samples from smokers secreted significantly lower levels of many inflammatory cytokines/chemokines. However, when stimulated with HSV-1, significantly higher levels of both PGE_2_ and many cytokines/chemokines were secreted from smokers’ blood samples than from controls. In terms of blood cell composition, red blood cells, hematocrits, hemoglobin levels, MCV, MCH, MCHC, Pct and RDW levels were all elevated in smokers, in keeping with their compromised lung capacity. As well, total leukocytes were significantly higher, driven by increases in granulocytes and monocytes. In addition, smokers had lower NK cells and higher Tregs than controls, suggesting that smoking may reduce the ability to kill nascent tumor cells. Importantly, there was substantial person-to person variation amongst smokers with some showing markedly different values from controls and others showing normal levels of many parameters measured, indicating the former may be at significantly higher risk of developing lung cancer.

## Introduction

Tobacco smoking leads to the premature death of 6 million people worldwide, primarily from lung cancer, chronic obstructive pulmonary disease (COPD) and cardiovascular disease (CVD)^1^. The average loss of life, compared with never-smokers, is 10–15 years and smokers start to suffer from tobacco related diseases such as cardiovascular disease, stroke and dementia approximately 10 years earlier than non-smokers^2,3^. Interestingly, however, only 15–20% of heavy smokers go on to develop lung cancer^4,5^. It would therefore be very helpful to identify those most susceptible to lung cancer early on so that preventative measures can be instituted. Since chronic inflammation (CI) has been shown to be a major player in lung cancer^6–9^, a number of studies have been carried out to correlate inflammatory biomarkers with the subsequent development of lung cancer. From these studies there is a general consensus that the acute phase protein, C-reactive protein (CRP), is a sensitive biomarker for cigarette smoke-induced inflammation^10–12^, and may be useful for risk prediction^13^. As well, plasma levels of the upstream regulators of CRP, interleukin-1β (IL-1β) and IL-6^14^ have been shown in a number of studies to be elevated in smokers^15,16^. Related to this, it was very recently reported that CRP and IL-6 levels were significantly higher among 10,061 atherosclerosis patients subsequently diagnosed with lung cancer than among those not subsequently diagnosed with lung cancer^17^. Importantly, these investigators found that reducing the inflammatory markers, CRP and IL-6 (and likely other pro-inflammatory proteins) with canakinumab (a monoclonal antibody against IL-1β) dramatically reduced the incidence of lung cancer in these patients^17^.

A number of studies have shown elevated levels of white blood cells in heavy smokers as well as CRP and another acute phase protein, fibrinogen^13,18^. One study has investigated all three and shown that simultaneously elevated CRP, fibrinogen and leukocyte count is associated with not only an increased risk of lung cancer but of colorectal and breast cancer as well^13^. As well, pre-diagnostic blood samples from ever-smokers who subsequently developed lung cancer were found, in a recent study, to have elevated levels of 4 proteins, cancer antigen 125 (CA125), carcinoembryonic antigen (CEA), cytokeratin-19 fragment (CYFRA 21-1) and the precursor form of surfactant protein B (Pro-SFTPB), suggesting this panel of biomarkers might be highly useful for smoking-induced lung cancer risk assessment^19^.

To explore the possibility of identifying new biomarkers that might be helpful in identifying heavy smokers that are at high risk of developing lung cancer we set out in the current study to (a) compare levels of CI, immune function and blood cell components in heavy smokers with controls and (b) compare results obtained from this cohort with our previously published data from our obese^20^ and normal aging^21^ cohorts.

## Results

### Smoking is associated with elevated CRP, fibrinogen, IL-6 and CEA but not PGE_2_ levels

**Figure 1:**
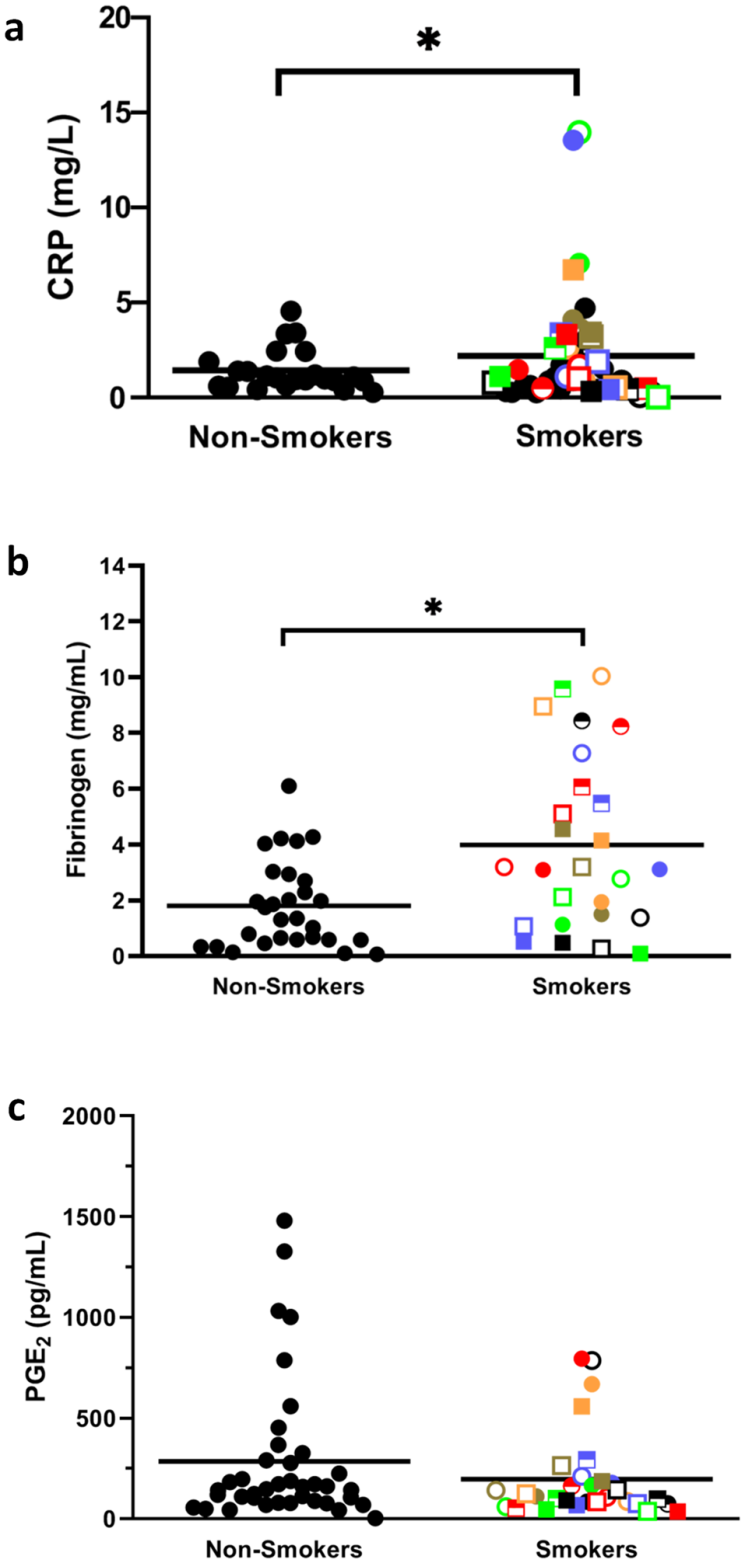
Smokers have elevated plasma levels of CRP and fibrinogen but not PGE_2_. Plasma samples were analysed for **(a)** CRP, **(b)** fibrinogen and **(c)** PGE_2_ ● = non-smoking, controls; All the smokers were given a unique identifying symbol so that each smoker could be tracked from one assay to another. As well, all males were symbolized by a square and all females by a circle. The mean is shown as a horizontal line within each group. * indicates a statistically significant difference (P < 0.05) between smokers and the non-smoking control group.

Thirty heavy smokers and 36 non-smokers (controls) were recruited for this study and their characteristics are shown in Table 1. To measure levels of CI, plasma samples were first analysed for CRP levels since this is a well-established marker for smoking-induced inflammation^10–13^. As shown in Fig. 1a, CRP levels were significantly (*p* = 0.046) elevated in the smokers. Of note, all the smokers were given a unique identifying symbol so that each smoker could be tracked from one assay to another. As well, all males were symbolized by a square and all females by a circle. We also compared fibrinogen^22^, another acute phase protein that has previously been shown to be elevated in smokers. As shown in Fig. 1b, fibrinogen levels were significantly (*p* = 0.005) elevated in the smokers as well but, as with CRP, there were many smokers with normal levels of fibrinogen. Interestingly, there was no correlation between the levels of these two proteins (R = 0.046) (Table 2, Supplementary Table S1). We next looked at PGE_2_ levels and found no significant (*p* = 0.259) increase in smokers (Fig. 1c).

**Table 1:** Characteristics of non-smoking controls (n = 36) and smokers (n = 30).

Characteristic	Non smokers	Smokers
Age (years)	58.4 ± 7.2	61.4 ± 5.1
Male %	33.3%	55.1%
BMI	22.7 ± 1.1	27.5 ± 7.8
COPD	–	31%
Pack years	–	45.9

**Table 2:**
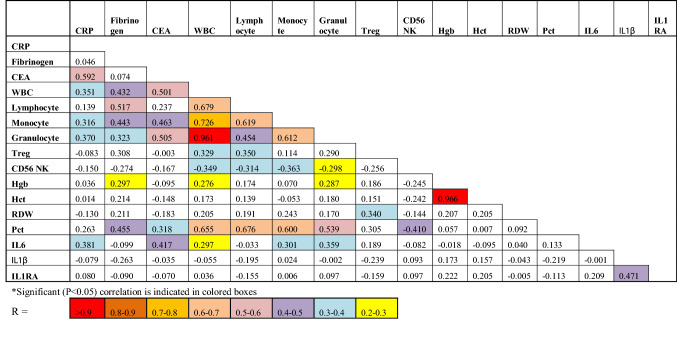
Pearson correlation values between baseline parameters*.

**Figure 2:**
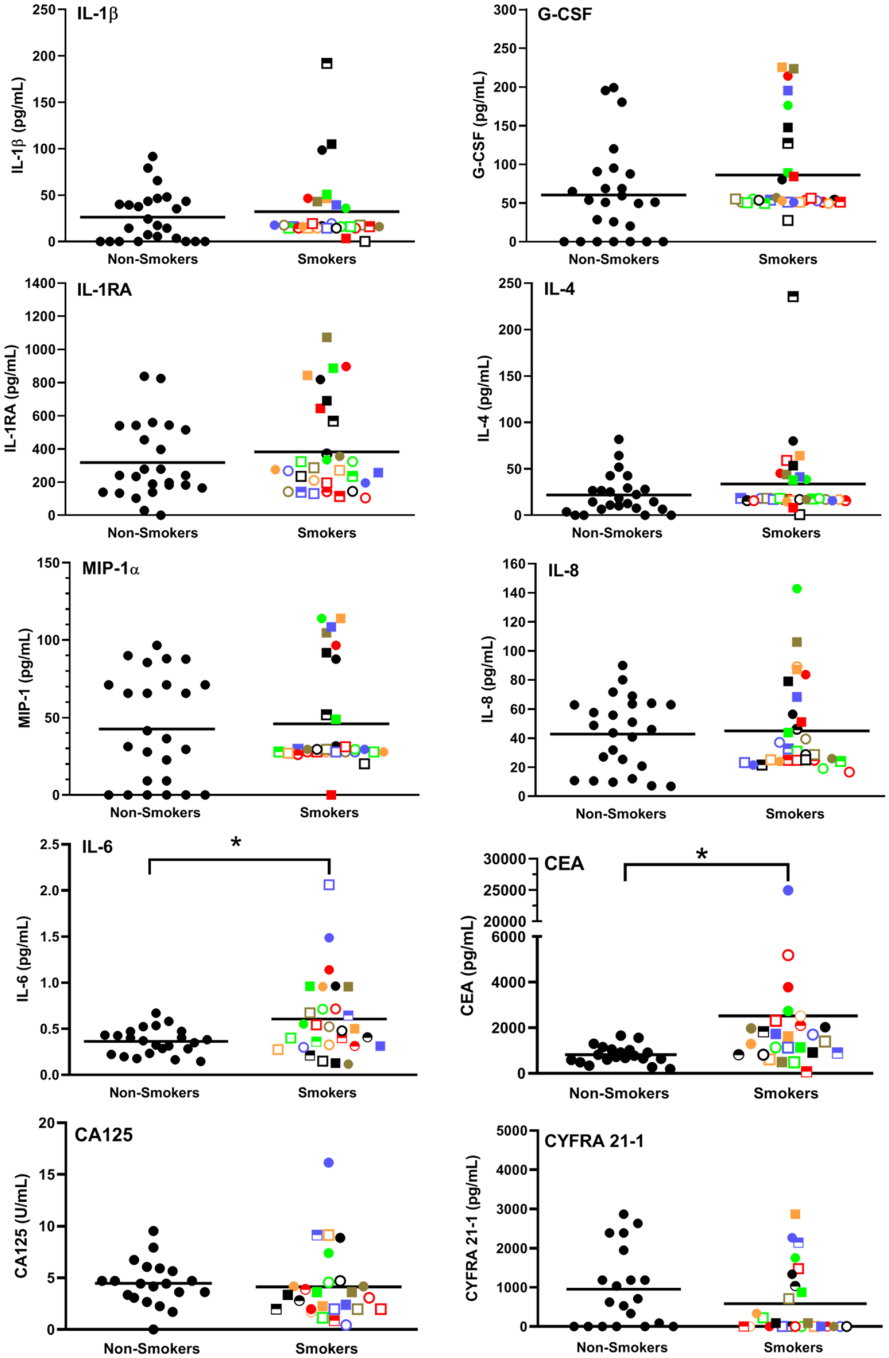
IL-6 and CEA are elevated in smokers. Plasma samples were analyzed for 15 cytokines/chemokines using Luminex technology. Only those cytokines/chemokines showing a trend towards different levels in smokers are shown. The mean is shown as a horizontal line within each group. * indicates a statistically significant difference (P < 0.05) between smokers and non-smoking controls.

We then measured the levels of IL-1β, G-CSF, IL-10, IL-13, IL-17, MIP-1α, VEGF, IFNγ, IL-12, IFNα, IL-1RA, TNFα, IL-4 and IL-8 using Luminex beads, and IL-6 via mesoscale analysis. While no differences were found in the levels of IL-10, IL-13, IL-17, VEGF, IFNγ, IL-12 or IFNα, there were trends, as shown in Fig. 2, towards elevated IL-1β, G-CSF, IL-1RA, IL-4, MIP1α and IL-8 in the plasma of smokers but only the level of IL-6 was significantly (*p* = 0.049) elevated. IL-6 levels, as expected, were significantly correlated with CRP (R = 0.381, *p* = 0.006), but no relationship was found with fibrinogen (R = -0.099) (Table 2). Given that IL-1RA competes with IL-1β for binding to the IL-1 receptor and prevents IL-1β-induced pro-inflammatory signalling we wondered if the increased production of IL-1RA in smokers was associated with high IL-1β levels. As shown in Table 2, there was indeed a significant (*p* = 0.0002), albeit only moderate correlation between IL-1β and IL-1RA levels (R = 0.471,). This was also demonstrated in Fig. 2, where 3 of the 4 subjects with the highest IL-1β levels (i.e.,  ,  ,  ) also had the highest IL-1RA levels. However, one subject with a high IL-1RA level ( ) had a very low IL-1β level, suggesting other factors may contribute to IL-1RA levels in these individuals.

As well, since a panel of biomarkers consisting of CEA, CA125, CYFRA 21–1 and Pro-SFTPB was recently found to be highly predictive of smokers subsequently developing lung cancer^19^, we were interested in determining their values. We did not test SFTPB, however, since there were no commercially available kits and it had been reported that SFTPB has a non-linear, J-shaped association with lung cancer risk (with no and high levels increasing risk), making interpretation of results difficult^23^. As shown in the last three panels of Fig. 2, only CEA was significantly (*p* = 0.0009) higher in smokers than non-smokers. Of interest, the levels of CEA were significantly correlated with those of CRP (R = 0.592, *p* = 0.00002) and IL-6 (R = 0.417, *p* = 0.004) but not with those of fibrinogen, IL-1β or IL-1RA (Table 2).

### Blood from smokers secrete elevated levels of inflammatory cytokines/chemokines when incubated with or without HSV-1 but reduced levels with *E. coli*

**Figure 3:**
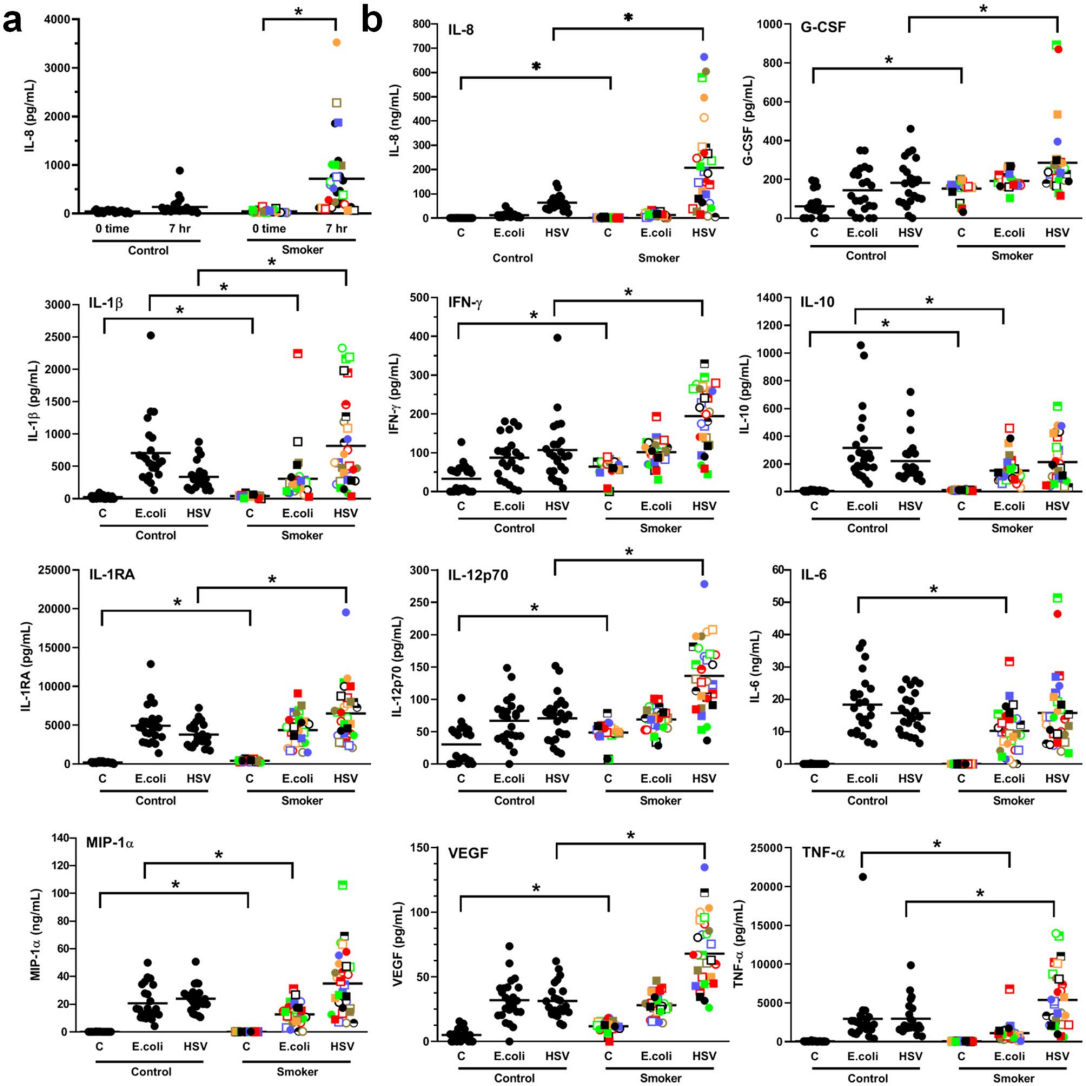
Incubation of whole blood samples from smokers yields higher levels of cytokines/chemokines in the presence or absence of HSV-1 but lower levels with *E. coli*. Whole fresh blood from smokers and controls were incubated for 7 h at 37 °C in the absence or presence of *E. coli* or HSV-1 and the conditioned plasma analyzed for the cytokine/chemokine levels using Luminex beads. **(a)** A comparison of plasma IL-8 levels from whole blood samples taken at time 0 or after 7 h of incubation in the absence of stimulation from smokers and controls. **(b)** A comparison of the plasma levels for 11 of the 15 cytokines/chemokines tested (selected on the basis of having a significant difference (P < 0.05) between smokers and controls). Shown are the levels obtained after 7 h of incubation without (**C**) or with stimulation by *E. coli* or HSV-1.

To determine if immune cells in the blood from smokers were more or less responsive to an ex vivo challenge, we incubated freshly isolated whole blood samples for 7 h (to obtain levels of both early and late cytokines^21^) in a 5% oxygen (to mimic in vivo conditions), humidified incubator at 37 ºC in the presence or absence of an immune cell challenge. To mimic an in vivo bacterial or viral infection, we employed intact *E. coli* and HSV-1 as in our earlier obesity^20^and aging studies^21^. As shown in Fig. 3a, blood samples from smokers incubated without any exogenous stimulus secreted significantly (*p* < 0.0001) higher levels of the neutrophilic chemokine, IL-8, than whole blood from controls. A similar, albeit less robust, trend to higher cytokines/chemokines in 7 h incubated, unstimulated blood samples from smokers was also seen with IL-1β, G-CSF, IL-13, IL-17, VEGF, IFNγ, IL-12, IFNα, IL-4 and MIP1α (Fig S1).

In response to HSV-1 stimulation, significantly elevated levels of G-CSF (*p* = 0.012), IL-8 (*p* = 0.002), IL-1β (*p* = 0.004), IFNγ (*p* = 0.0002), IL-1RA (*p* = 0.0005 ), IL-12 (*p* < 0.0001), TNFα (*p* = 0.003) and VEGF (*p* < 0.0001), were observed with whole blood from smokers (Fig. 3b). However, in response to *E. coli* stimulation this trend was reversed with significantly lower levels of IL-1β (*p* = 0.002), IL-10 (*p* = 0.0003), IL-6 (*p* = 0.0004), MIP1α (*p* = 0.02) and TNFα (*p* < 0.0001) in whole blood samples from smokers than from controls (Fig. 3b). Of note, there was a strong correlation, following *E. coli* stimulation, between IL-6 and MIP-1α (R = 0.913) levels as well as between IL-1β and TNFα (R = 0.822) levels (Table 3, Supplementary Table S2).

**Table 3:**
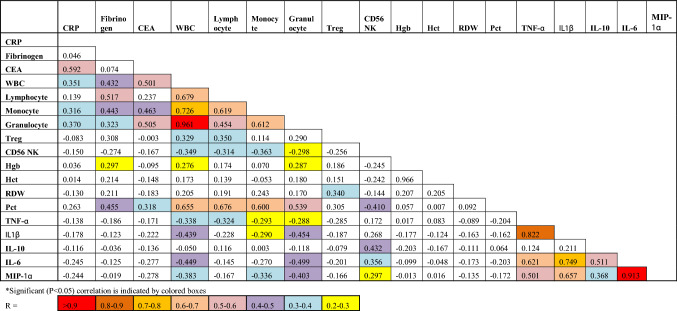
Pearson correlation values between parameters after *E. coli* stimulation of whole blood.

**Figure 4:**
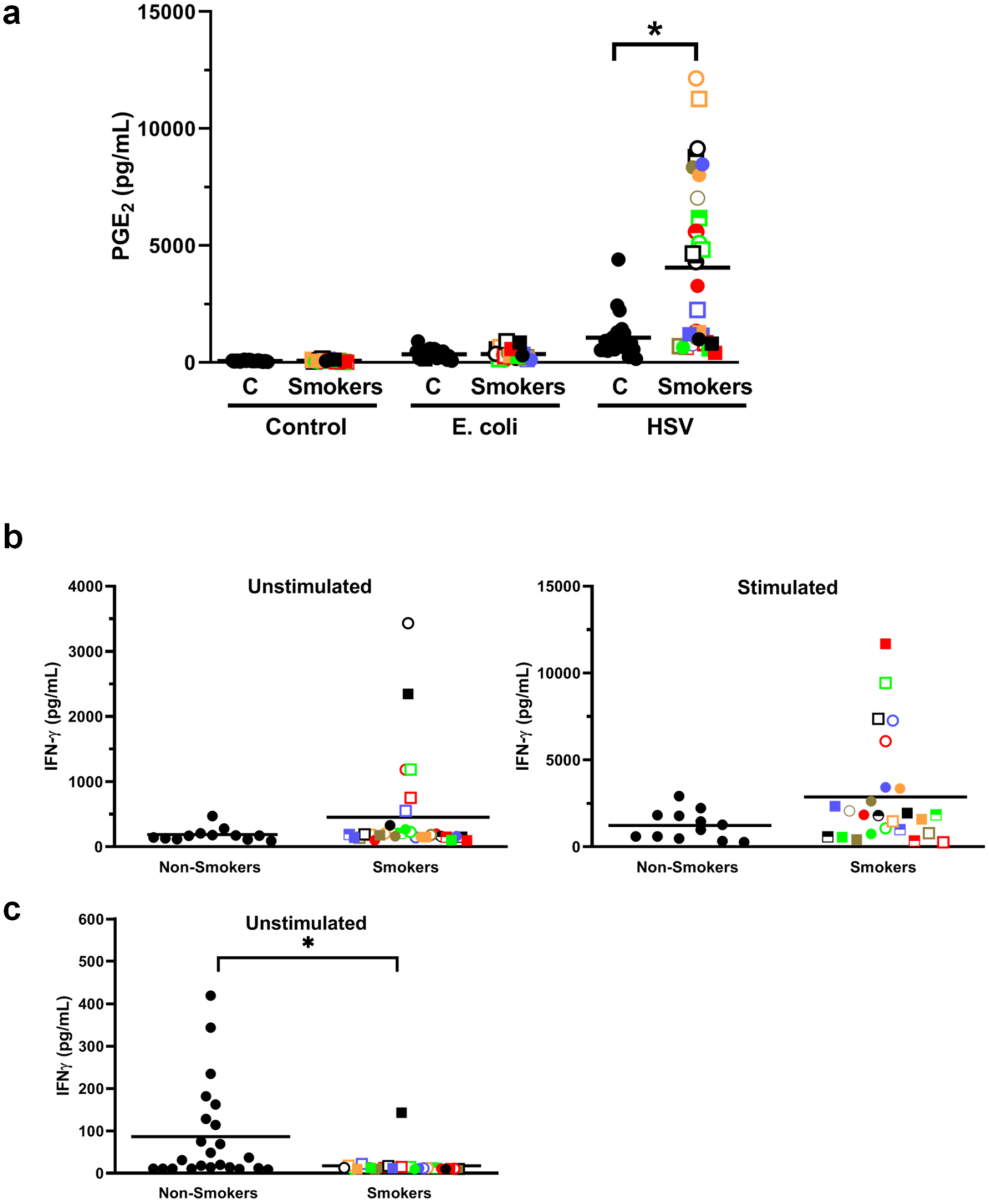
Whole blood samples from smokers secrete significantly more PGE_2_ in response to HSV-1 stimulation and more IFNγ in response to T cell stimulation than controls. **(a)** Whole fresh blood from smokers and controls were incubated for 7 h at 37 °C ± *E. coli* or HSV-1 and the conditioned plasma analyzed for PGE_2_ levels. **(b)** Whole fresh blood from smokers and controls were diluted tenfold with RPMI and incubated for 4 days at 37 °C in the absence (left panel) or presence (right panel) of anti-CD3 + anti-CD28 antibodies. **(C)** PBMCs from smokers and controls were incubated without T cell stimulation for 4 days in RPMI + 10% autologous plasma and the levels of IFNγ production determined. Each smoker is identified with a unique symbol. * indicates a statistically significant difference (P < 0.05) between controls and smokers.

Interestingly, although endogenous levels of PGE_2_ were not significantly (*p* = 0.259) increased in smokers (i.e., at zero time) (Fig. 1c) or after 7 h of incubation without an exogenous stimulus, there was a dramatic increase in the levels of this eicosanoid in response to HSV-1 in the blood from smokers (Fig. 4a). This was not seen with *E. coli* stimulation. This novel biomarker for smokers correlated moderately with fibrinogen (R = 0.461) and strongly with HSV-1-stimulated VEGF (R = 0.778), IL-8 (R = 0.725), IFNγ (R = 0.613), IL-12 (R = 0.751) and IL-1RA (R = 0.567) (Table 4, Supplementary Table S3).

**Table 4:**
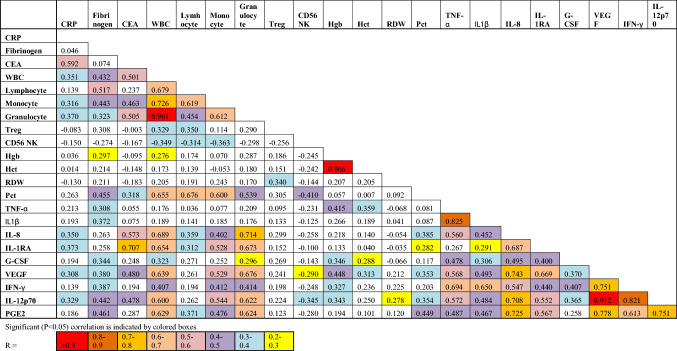
Pearson correlation values between parameters after HSV-1 stimulation of whole blood.

### Smoking tends to increase IFNγ secretion from activated T cells

To evaluate the effect of smoking on T cell activation, fresh whole blood from smokers and controls were diluted tenfold with RPMI as described^24^ and stimulated or not with anti-CD3 + anti-CD28 for 2 days. As shown in Fig. 4b, there was a trend towards higher levels of IFNγ secretion under both unstimulated and stimulated conditions. When the same experiment was carried out with isolated PBMCs (in 10% autologous plasma to simulate in vivo conditions) instead of whole diluted blood, anti-CD3 + anti-CD28 stimulation for 4 days also resulted in a trend towards higher levels in smokers (Fig S2). However, in the absence of T cell stimulation there were significantly (*p* = 0.007) lower levels of IFNγ in the smokers (Fig. 4c) rather than higher levels when whole blood was used (Fig. 4b). This difference suggests that further studies are needed to determine which of these in vitro assays more accurately reflects in vivo conditions.

### Smoking increases the size and hemoglobin level of red blood cells

**Figure 5:**
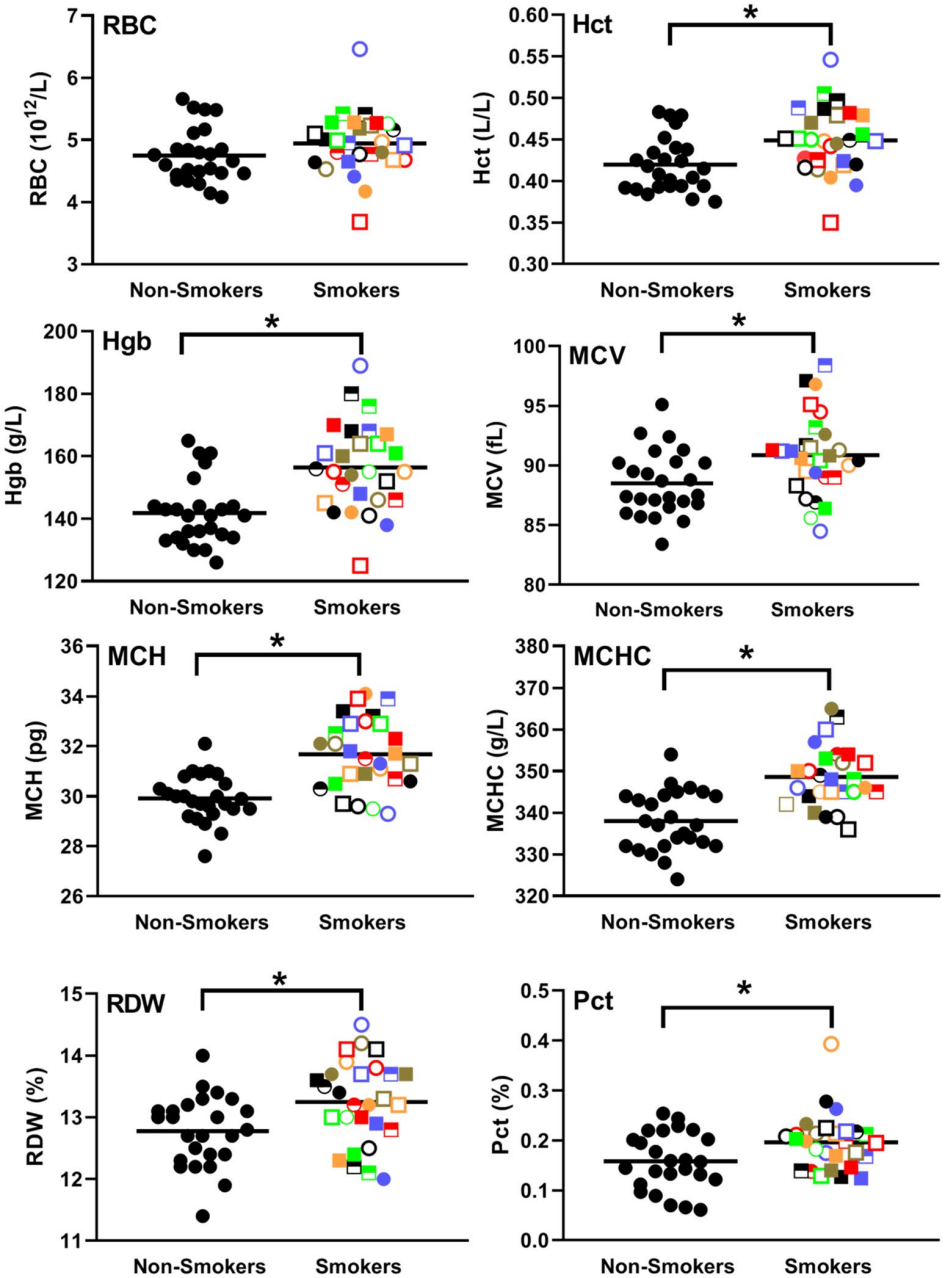
Smoking increases hematocrits, HgB, MCV, MCH, MCHC, RDW and Pct but not RBC levels. Smokers were compared to controls for RBC, Hct, HgB, MCV, MCH, MCHC, RDW and Pct. Each smoker is identified with a unique symbol. * Indicates a statistically significant difference (P < 0.05) between smokers and non-smoking controls.

Blood differential cell counts revealed that while there was no significant (*p* = 0.212) difference in the number of red blood cells (RBCs) between smokers and controls (top left panel, Fig. 5), hematocrits (top right panel, Fig. 5, *p* = 0.01) and hemoglobin levels (2nd row left panel, Fig. 5, *p* = 0.0002) were significantly elevated in smokers. As well, MCV, which indicates the average volume of each RBC, MCH, which indicates the average mass of hemoglobin/rbc and MCHC, which indicates the average concentration of hemoglobin/RBC, were all significantly(*p* < 0.05) elevated in smokers (Fig. 5). Taken together these results demonstrate that rbcs in smokers are larger and have more hemoglobin/RBC than in non-smoking controls. Also of note, RBC distribution width (RDW) was significantly (*p* = 0.007) higher in smokers (bottom left panel, Fig. 5). Pearson analyses revealed little to no correlation between any of these rbc markers and CRP or fibrinogen (Table 2), suggesting that they may be useful independent biomarkers for risk assessment. We also found that while there was only a slight trend towards a higher platelet count in smokers (Fig S3), the plateletcrit (Pct, i.e., total platelet mass) was significantly (*p* = 0.02) elevated in smokers (bottom right panel, Fig. 5). Pearson analyses suggested a moderate correlation between smoker plateletcrit and fibrinogen levels (R = 0.455) (Table 2).

### Smokers have elevated monocytes, granulocytes and Tregs and reduced NK cells

**Figure 6:**
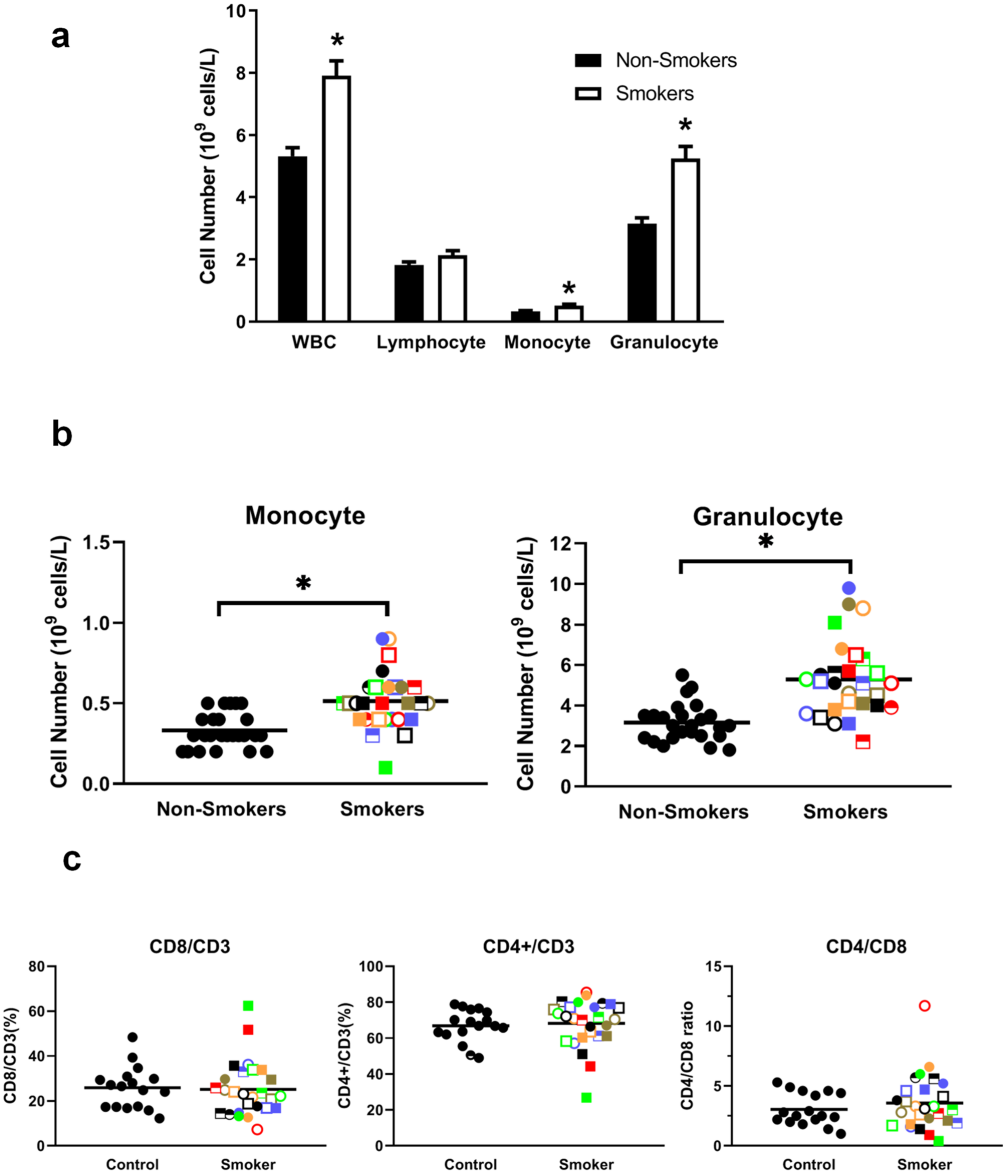
Smoking increases monocytes and granulocytes but does not affect CD4 and CD8 T cell populations. Smokers were compared with non-smokers for **(a)** total white blood cells, lymphocytes, monocytes and granulocytes. Results are expressed as the mean ± SEM. **(b)** Smokers were compared with non-smokers for monocytes and granulocytes. **(c)** Smokers were compared with non-smokers for CD4 + and CD8 + T cells and their CD4/CD8 ratio. Each smoker is identified with a unique symbol in panels B and C. * indicates a statistically significant difference (P < 0.05) between smokers and non-smokers.

Flow cytometry was also carried out to look for differences in the proportion of various immune cells between smokers and controls. While there were no significant (*p* = 0.058) differences in lymphocytes (Fig. 6a), there was a significant (*p* < 0.0001) increase in total white blood cells in smokers and this was attributable to a significant increase in monocytes (*p* < 0.0001) and granulocytes (*p* < 0.0001) (Fig. 6a). Pearson analyses revealed that both monocyte and granulocyte numbers correlated with CRP levels (R = 0.316 and 0.370, respectively) and with CEA levels (R = 0.463 and 0.0.505, respectively). In addition to monocyte and granulocyte numbers, lymphocyte numbers were also correlated with fibrinogen (R = 443, R = 0.323 and R = 0.517 respectively) (Table 2). Figure 6b shows the levels of monocytes and granulocytes for each smoker so that they could be compared with other biomarkers. Of interest, while most smokers with high granulocytes also had high monocytes, one smoker with a high granulocyte level ( ) had a very low monocyte level suggesting, at least in part, independent regulation. Pearson analysis confirmed only a moderate correlation between monocyte and granulocyte levels (R = 0.612) (Table 2).

**Figure 7:**
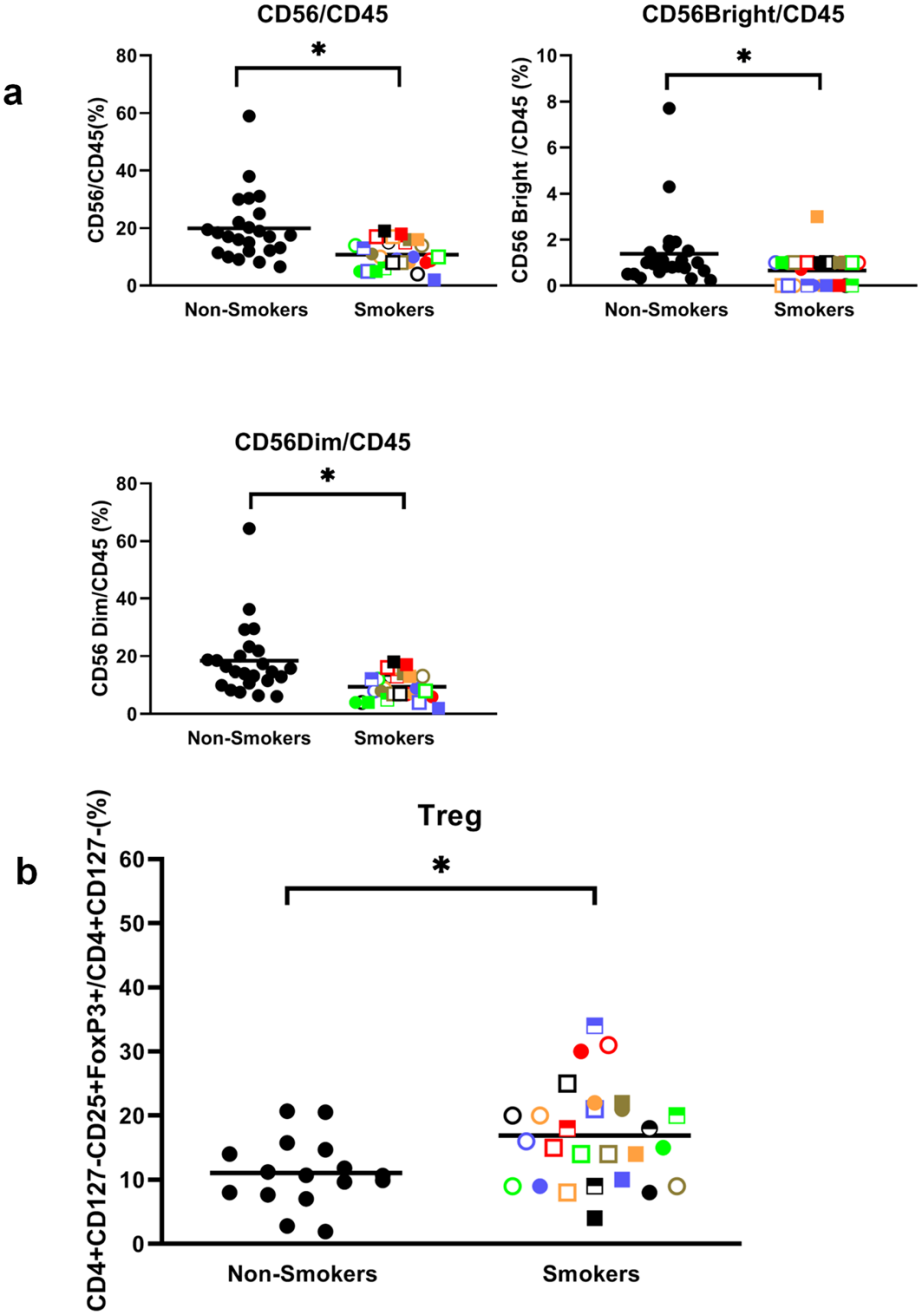
Smoking reduces natural killer cells and increases regulatory T cells. (**a**) Smokers and non-smokers were compared for CD56/CD45, CD56Bright/CD45 and CD56Dim/CD45 populations. **(b)** Smokers and non-smokers were compared for Treg levels. Each smoker is identified with a unique symbol. * indicates a statistically significant difference (P < 0.05) between smokers and controls.

As shown in Fig. 6c, smokers did not show any changes in CD4 + or CD8 + T cells and thus no change in their CD4/CD8 ratio (Fig. 6c). This contrasts strongly with our previous studies showing people with BMIs > 35 display a reduction in CD8 + and an increase in CD4 + T cells such that their CD4/CD8 ratio is more than double that of controls^20^. On the other hand, similar to our obesity study we found a significantly (*p* = 0.0002) lower proportion of natural killer (NK) cells (i.e., both CD56 bright and dim cells) and higher Tregs (CD4 + CD127low/-CD25 + FoxP3 + cells) (Fig. 7), suggesting a potentially poorer ability to kill cancer cells. Pearson analyses showed no correlation between smoker NK cell numbers and either CRP or fibrinogen levels (Table 2). As well, there was no correlation between Tregs and CRP (R = − 0.083) and only a moderate correlation between Tregs and fibrinogen (R = 0.308) (Table 2), suggesting both NK cell and Treg numbers may be independent biomarkers of cancer risk.

## Discussion

### Smoking is associated with elevated CRP, fibrinogen, IL-6 and CEA levels

CRP and fibrinogen are classified as positive acute phase proteins, which means their production and secretion are upregulated in the liver during inflammation. They, as well as other positive acute phase proteins, have both pro- and anti-inflammatory properties and modulate immune cells to destroy infectious agents, remove damaged tissue and promote repair. Smoking as well as obesity and CVD have been associated with elevated CRP and fibrinogen levels^22,25,26^. While the physiological impact of elevated CRP has not as yet been fully resolved^14,27^, elevated fibrinogen levels are thought to lead to platelet aggregation, increased blood viscosity, thrombosis and CVD, in part via its conversion into fibrin during blood coagulation^8,13^. This increase in plasma fibrinogen levels in smokers is thought to be a general inflammatory response to persistent insult by cigarette smoke^28^, typical in a wound healing process. One of the main mediators that may stimulate this response is IL-6^28^, which we demonstrate in this study to be elevated in smokers.

Even though CRP and fibrinogen are both upregulated in response to IL-6^29^ we only observed a moderate correlation between IL-6 and CRP levels (R = 0.381) and saw no significant (*p* = 0.50) correlation with fibrinogen levels (R = − 0.099), suggesting that other factors determine levels of these acute phase proteins. Catecholamines and free fatty acids for example, have been reported to also stimulate the synthesis of fibrinogen^22,28^ and are elevated after smoking.

Also of note, while it is now established that IL-1β is upstream of IL-6^10,14^ we found that IL-1β levels showed no correlation with IL-6 or CRP levels (R = − 0.001 and − 0.079, respectively) nor with fibrinogen levels (R = − 0.263). These results might be explained, at least in part, by the recent finding that CRP acts to downregulate its own production via inhibition of IL-1β production from monocytes and that this, in turn, reduces IL-6 production^14^. Of note, our finding that endogenous plasma levels of PGE_2_ were not elevated in smokers was surprising given previous reports of elevated PGE_2_ levels in smokers^30,31^. They were also surprising given our results showing increased plasma PGE_2_ levels in obese subjects^20^, another group prone to CI.

Luminex analyses of fresh blood samples revealed a trend towards higher levels of IL-1β, G-CSF, IL-1RA, IL-4, MIP1α, IL-8 and IL-6 in smokers but this reached statistical significance only with IL-6. This prototypical pro-inflammatory cytokine is a well-established marker of CI^32,33^ and not only triggers the acute phase protein response, which includes the release of fibrinogen and CRP from hepatocytes, but also stimulates antibody production and the differentiation of TH_17_ and CD8 + T cells and reduces the differentiation of Tregs^34^. Taken together, this, in turn, leads to CI. Thus, the elevated endogenous levels of IL-6 that we and others have observed^35,36^ may play a role in the increased lung cancer risk to which smokers are prone^1^. The trend towards higher G-CSF levels that we observed in smokers has been reported previously^37^ and is of potential import since the administration of this cytokine to humans has been shown to increase both granulocytes and monocytes^38^ and thus may be at least partially responsible for the increase we see in these white blood cells in smokers.

Luminex analyses of fresh blood samples also revealed that of three previously established biomarkers of lung cancer risk, i.e., CEA, CA125, CYFRA 21-1^19^, only CEA was higher in smokers. Interestingly, Pearson correlation analyses (Table 2) revealed that CEA levels correlated with monocyte (R = 0.463) and granulocyte (R = 0.505) numbers.

Of note, while the literature suggests that smokers with COPD may be more inflamed than smokers without COPD^39–41^ we did not see any substantial differences in IL-6, CRP, VEGF or IL-17 levels between the 9 smokers with COPD and the 21 non-COPD smokers. However, this may be attributable to our small sample sizes. On the other hand we did find that those with COPD had significantly increased granulocytes and, in response to HIV-1 stimulation, significantly lower levels of IL-1β.

We also compared male and female smokers in all assays and, unlike Melanica et al. (2017), found no significant sex-dependent differences in hematological parameters^51^ which could be also due to a much smaller sample size when compared to that used by Melanica et al. (2017). However, we did find that female smokers had significantly higher CEA levels and higher IL-1β levels from HSV-1 stimulated blood. On the other hand, we found that male smokers had a significantly higher CD8/CD3 T cell ratio and higher VEGF, TNFα and MIP1α from *E. coli*-stimulated blood.

### Smoking alters innate and acquired immune responses

A novel finding in our study is that simply incubating fresh whole blood from smokers for 7 h without any exogenous stimulus increases the levels of a number of pro-inflammatory cytokines/chemokines, the most robust being IL-8. This ex-vivo generation of pro-inflammatory cytokines may be related to the higher levels of oxidative stress reported in cigarette smokers^42,43^. Cigarette smoking generates many reactive species, which may overwhelm the antioxidant defense system. While low molecular weight antioxidants such as vitamin E and C have been reported to be lower in smokers^42^, blood glutathione levels are 8–10% higher^44^. It is thus possible that cigarette smokers produce a compensatory level of glutathione to maintain a comparable in vivo level of cytokines/chemokines to non-smokers. However, ex vivo this may not be the case, perhaps because of limiting cysteine levels and this, potentially, may be the reason we observe increased pro-inflammatory cytokines being generated ex vivo from the blood of smokers.

Of the 15 cytokines/chemokines examined, following 7 h of stimulation with *E. coli*, we found a number were lower in smokers, suggesting, perhaps, a compromised ability to eradicate bacterial infections. This is consistent with the well-known increased susceptibility of smokers to infection. However, this increased susceptibility is also likely due in part to the deleterious effects of cigarette smoke on the integrity of the respiratory epithelium, which provides easy entry for pathogens^45,46^.

Stimulation with HSV-1, on the other hand, revealed a more robust cytokine/chemokine response in the blood of smokers, perhaps suggesting an increased susceptibility to a cytokine storm. Related to this, we did not see an increase in HSV-1 stimulated IFNα, an important cytokine involved in limiting viral infections^47^. As well, we observed a marked increase in PGE_2_ in response to HSV-1 stimulation in smokers. Since PGE_2_ has been shown to inhibit both B and T cell differentiation and activation^48,49^, this could result in a reduced ability to clear viral infections. This is consistent with the generally accepted notion that cigarette smoking is, in general, immunosuppressive^50^. Together with the more robust inflammatory response we observed to a viral challenge, this may explain why viral infections tend to be more severe in smokers^51^ and may serve as a caution when treating lung cancer patients with oncolytic viral therapy.

### Smoking and blood cells

Our finding that smoking increases the size and hemoglobin level of rbcs is consistent with the literature^52,53^ and is in keeping with carbon monoxide in tobacco smoke displacing oxygen from hemoglobin^54^. The increased RDW that we observed in smokers may be a useful cancer risk marker since a high RDW has been associated with metabolic syndrome, CVD and all-cause mortality^55^. The increased plateletcrit that we observed in smokers, in the absence of a increase in platelet numbers, may also be helpful in assessing risk since this measure of total platelet mass has recently been shown to be useful as a biomarker for various inflammatory-related conditions, including Crohn’s disease and gestational diabetes^56,57^. Thus, the higher plateletcrit that we observed in smokers may suggest that this underutilized, yet readily available biomarker warrants further investigation as a possible biomarker for cancer risk in smokers.

Although our finding that lymphocytes are not elevated in smokers contrasts with some recent reports^18,58^, our finding that granulocytes and monocytes are elevated in smokers is consistent with the literature^59^. This may be due, in part, to the elevated endogenous levels of G-CSF we observed in the plasma of smokers, perhaps as a result of cigarette smoke-induced damage to bronchial epithelial cells^60^, since this increases myelopoiesis. G-CSF has also recently been suggested as an orchestrator of inflammation in COPD^61^, and has been found to be elevated in the bronchoalveolar lavage fluid of smokers^61^. Since COPD increases the risk of lung cancer^62^, it is possible that an increase in G-CSF in smokers could be a biomarker of future cancer risk. In addition to promoting myelopoiesis, G-CSF also appears to improve the survival of neutrophils in the lungs^60^. Importantly, higher neutrophils have been shown to correlate with poor survival for non-small cell lung cancer patients^63–65^ and it is therefore possible that this may be a predictor of increased risk of future lung cancers in smokers.

In addition to increased monocytes and neutrophils, we also observed higher peripheral Tregs and lower NK cells in smokers. Elevated Tregs, which down regulate immune responses, have been reported previously in smokers^66,67^ and may serve as a counterbalance to the CI triggered by smoking. The reduction in NK cells we observe may be due to reduced NK proliferation in the bone marrow or increased loss of NK cells during entry into tissues or a shortened half-life^68^. These changes in Tregs and NK cell levels not only signal an imbalance in homeostasis but they may also foster an environment favourable to cancer growth by reducing immune cell killing of nascent tumor cells. As such, they may still serve as valuable biomarkers for increased risk of future lung cancer.

### A comparison of the effects of smoking and obesity on inflammatory biomarkers

Heavy smokers, like those with BMIs > 35, have elevated blood levels of CRP, although these levels are higher in obesity (mean = 10 µg/ml vs 3 µg/ml in smokers^20^, in keeping with earlier studies showing CRP levels are most sensitive to BMI changes^69^. As well, like those with BMIs > 35, smokers have elevated Tregs, granulocytes and monocytes and reduced numbers of NK cells. However, unlike smokers, those with BMIs > 35 have elevated CD4/CD8 ratios^20^. Our finding that smoking does not affect CD4 or CD8 T cell levels or the CD4/CD8 ratio is at odds with several studies, reviewed in^70^, but is consistent with^71^. It thus appears that the currently available data on CD4 and CD8 frequency in smokers is conflicting. Smoking intensity may contribute to the discrepancy, since peripheral blood T cell subsets were not found to be different in light (10–19 pack years) to moderate smokers (20–49 pack years)^71,72^, but a decrease, rather than an increase, in CD4/CD8 was observed in heavy smokers (50–120 pack years)^72,73^. In contrast to those with BMIs > 35, we also found that heavy smokers do not have elevated endogenous PGE_2_ levels in their plasma compared to controls^20^.

### Individual variation amongst smokers and subsequent risk of cancer

Having a unique symbol for each of the smokers has enabled us to track each individual through the different assays and determine, to some degree, their relative risk. An important caveat to our study, however, is that our sample size is relatively small and our data set has only been derived from a single time point. As well, what is difficult to determine at this time is the relative weight to be given to the different biomarkers, including those that may counter CI. Expanding our sample size and following these people over time to see who develops lung cancer, as we intend to do, should help to clarify this.

## Methods

### Human subjects and blood collection

Thirty heavy smokers (defined as current smokers smoking ≥ 1 pack/day for at least 20 years) and 36, healthy never smokers volunteers were recruited. Blood was collected into one K2 EDTA Vacutainer tube (Cat. No. 367861, BD, Mississauga, ON) and one endotoxin-free^74^ glass sodium heparin Vacutainer tube (cat. no. 366480, BD, Mississauga, ON). All participants gave informed written consent to participate in these studies. All experiments were performed in accordance with guidelines/regulations which were reviewed and approved by the joint Clinical Research Ethics Board of the University of British Columbia and BC Cancer (#H12-00727). All volunteers were asked to refrain from consuming non-steroidal anti-inflammatory drugs for 2 days prior to their blood draw. All blood samples were collected by trained phlebotomists at BC Cancer between 8:30 am and 10:00 am to avoid reported changes in cytokine secretion with diurnal rhythms^75^.

### Human blood assay

Whole blood assay was performed as previously described^20^. Human blood samples collected in sodium heparin containing glass tubes were mixed gently, kept at 23 °C and aliquoted within 2 h of collection into 96-well round bottom tissue culture plates. 50 μL of blood was added to individual wells along with 10 μL of either PBS (Control), *Escherichia coli* (*E. coli*, One Shot INV 110, Life Technologies, Burlington, ON) at a final concentration of 2 × 10^4^ cells/mL or HSV-1 G207 at a multiplicity of infection (MOI) of 0.06 (relative to total white blood cell numbers). Plates were then incubated for 7 h in a 5% oxygen, humidified incubator at 37ºC. Following incubation, 100 µL of PBS was added to each well, the cells were then thoroughly resuspended and centrifuged at 424 × *g* at 4ºC for 5 min. Supernatants were collected and immediately frozen at − 80ºC.

### Luminex analysis

A custom magnetic Luminex assay panel from Life Technologies was used as previously described^20^ to assess the levels of the following 15 cytokines and chemokines in human plasma: IL-1β, G-CSF, IL-10, IL-13, IL-6, IL-17, MIP1α, VEGF, IFNγ, IL-12p70, IFNα, IL-1RA, TNFα, IL-4 and IL-8. The cancer biomarkers CA125, CEA and CYFRA21-1 were measured using a Milliplex MAP Human Circulating Cancer Biomarker Panel from EMD Millipore. Frozen samples were thawed and centrifuged before testing (1000 × g at 4 ºC for 10 min). Plasma samples were incubated with antibody beads overnight at 4 ºC. Assay plates were read using a BioPlex 100 instrument utilizing Bio-Plex Manager 6.0 software (Bio-Rad Laboratories, Mississauga, ON).

### MesoScale analysis

Undiluted plasma samples were analyzed using a Mesoscale Discovery (MSD) V-PLEX Pro-inflammatory Panel 1 (human) kit for IL-6 quantification and Cytokine Panel 1 (human) kits for IL-17 and VEGF quantification (K15049D & K15050D, Mesoscale Discovery, Gaithersburg, MD) according to the manufacturer’s instructions and as previously described^20,76^. Briefly, samples were incubated on the MSD plates for 2 h at 23 °C with shaking. Plates were washed and incubated an additional 2 h with detection antibodies. After washing, 2 × Read buffer T was added to each well and the plate analyzed in a QuickPlex SQ 120 model no. 1300. Calibrator and plasma samples were analyzed in duplicates. Using the MSD Workbench software the response of the calibrator concentrations was plotted as log signal unit on the vertical (Y) axis versus log concentration on the horizontal (X) axis.

### PGE_2_, fibrinogen and CRP measurements

ELISAs for PGE_2_ (Cat #514010, Cayman Chemical Company, Ann Arbor, MI), fibrinogen (cat# ab208036, Abcam) and CRP (Cat # DCRP00, R&D Systems, Minneapolis, MN)^21^ were performed according to the manufacturers’ instructions.

### Blood differential counts

Blood differential cell counts were carried out on fresh whole blood collected in EDTA rather than heparin tubes to avoid heparin-induced aggregation of platelets^77^, using a Coulter Ac•T diff2™ Hematology Analyzer (Beckman-Coulter Corp., Miami, FL).

### Immunophenotyping

Human peripheral blood mononuclear cells (PBMCs) were isolated from heparinized whole blood by density gradient centrifugation with Lymphoprep (StemCell Technologies, Vancouver, BC) and immunophenotyped as previously described^21^. The PBMCs were stained with GhostDye Violet 450 viability dye (Tonbo Biosciences, San Diego, CA) for 30 min at 4ºC, washed once with PBS containing 2% FBS and 0.05% sodium azide (PFN), and blocked with anti-human CD32 Clone IV.3 (StemCell Technologies, Vancouver, BC) for 15 min at 23 °C. This was followed by staining of cell surface markers for 30 min at 23 °C. The cells were then washed twice and resuspended in PFN followed by flow cytometric analysis. To identify regulatory T cells, cells were fixed and permeabilized using the FoxP3 Staining Buffer Set (eBioscience, San Diego, CA). The cells were stained with the FoxP3 antibody overnight at 4ºC, washed once with PFN and analyzed by flow cytometry. All analysis was performed using a BD LSR Fortessa flow cytometer (BD Biosciences) and data analysis was performed using FlowJo software V10.2 (FlowJo, Ashland, OR). The antibodies used were: CD8-PE (clone SK1) and CD3-FITC (clone SK7) from StemCell Technologies, Vancouver, BC; CD45-FITC (Hle1), CD28-APC (clone CD28.2), CD4-PE-Cy7 (clone SK3), CD25-BB515 (clone 2A3), CD127-AF647 (clone HIL-7R-M21) and FoxP3-PE (clone 236A/E7) from BD Biosciences, Mississauga, ON; CD56-APC (clone CMSSB) from eBioscience, San Diego, CA.

### T cell activation

Fresh whole blood samples collected in heparin were diluted tenfold with RPMI and 90 µl of the diluted blood was incubated in 96-well flat-bottom tissue culture plates in a humidified incubator at 5% CO_2_, 37ºC for 2 days in the presence or absence of anti-CD3 + anti-CD28. Specifically, to stimulate the T cells the plates were pre-coated overnight with 2 µg/mL of anti-human CD3 (clone OKT3, eBioscience, San Diego, CA). Anti-human CD28 (clone CD28.2, eBioscience, San Diego, CA) was then added at a final concentration of 1 µg/mL to each well. Alternatively, PBMCs isolated as for immunophenotyping above were stimulated anti-CD3 and anti-CD28 as previously described^21^. PBMCs were counted using a Vi-Cell XR cell viability analyzer (Beckman Coulter, Brea, CA) and resuspended at 10^6^ cells/mL in RPMI + 10% autologous plasma + 100 U/mL penicillin/streptomycin. The PBMCs were aliquoted (50 μl/well) into 96-well flat-bottom tissue culture plates pre-coated or not as above with 0.5 µg/mL of anti-human CD3 and then 2 µg/mL anti-human CD28 added. After four days of incubation at 37 ˚C, 5% CO_2_, the plates were then centrifuged at 300 × g in a Beckman TJ-6 centrifuge for 5 min and the supernatants collected for IFNγ analysis.

### Statistical analysis

Kolmogorov–Smirnov test and F-test for equality of variances was performed to evaluate the normality and homogeneity of variance of the data sets using Graphpad prism 8. Significant differences between the means of the cytokine/chemokine levels in the smokers versus non-smokers at time zero (i.e., to measure levels of CI), and in *E. coli* or HSV-1-stimulated blood samples and other end-point measures were evaluated using unpaired two-tailed t-tests when variances between the two groups were found to be equal and the normality assumption is met. Otherwise, the non parametric Mann–Whitney tests were performed. In addition, a Pearson correlation analysis between cytokines/chemokines, blood profile and other end point measures was performed using Graphpad Prism 8. P < 0.05 is considered as statistically significant.

## Supplementary information

Supplementary Information.
