# Supplementary material for: The effect of smoking on chronic inflammation, immune function and blood cell composition
Source: Sci Rep. 2020 Nov 10;10:19480. doi: 10.1038/s41598-020-76556-7 (PMC7655856; doi:10.1038/s41598-020-76556-7)
Supplement: Supplementary file 1 — Supplementary Information. [file 41598_2020_76556_MOESM1_ESM.pdf]

Supplementary Information

The effect of smoking on chronic inflammation, immune function and blood cell composition

Ingrid Elisia[1], Vivian Lam[1], Brandon Cho[1], Mariah Hay[1], Michael Yu Li[1], Michelle Yeung[1],

Luke Bu[2], William Jia[2], Nancy Norton[3], Stephen Lam[3], Gerald Krystal[1*]

[1]The Terry Fox Laboratory, BC Cancer,
Vancouver, British Columbia, Canada

[2]Brain Research Centre, University of British Columbia,
Vancouver, British Columbia, Canada

[3]Department of Integrative Oncology, British Columbia Cancer Research Center,
Vancouver, British Columbia, Canada

[*]Corresponding author

E-mail: gkrystal@ bccrc.ca (GK)

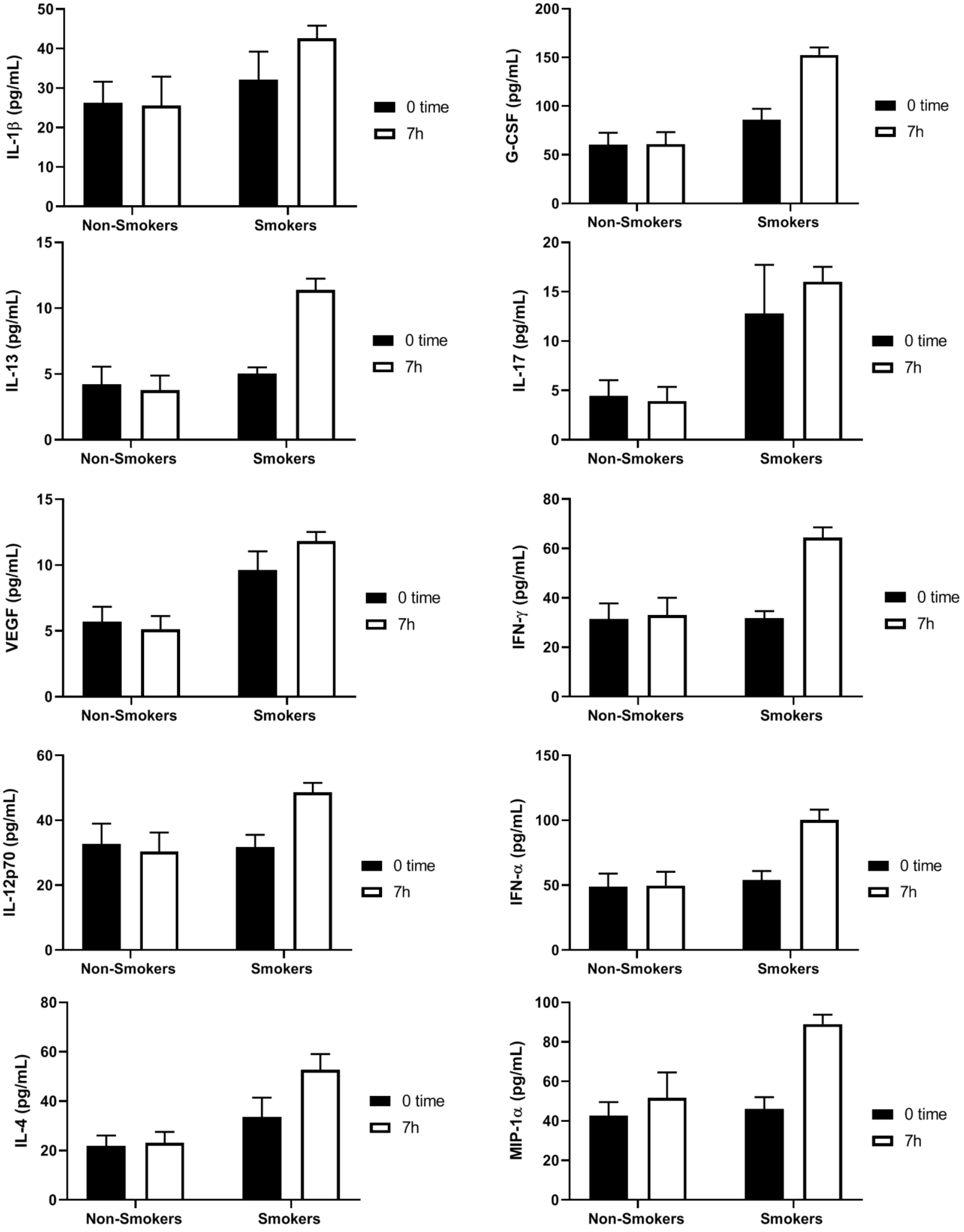

Supplemental Figure S1. Incubation of blood samples from smokers generates higher cytokines/chemokines than non-smokers. Unstimulated blood was incubated for 7h at 37°C and cytokines/chemokines in the plasma were quantified using luminex assay.

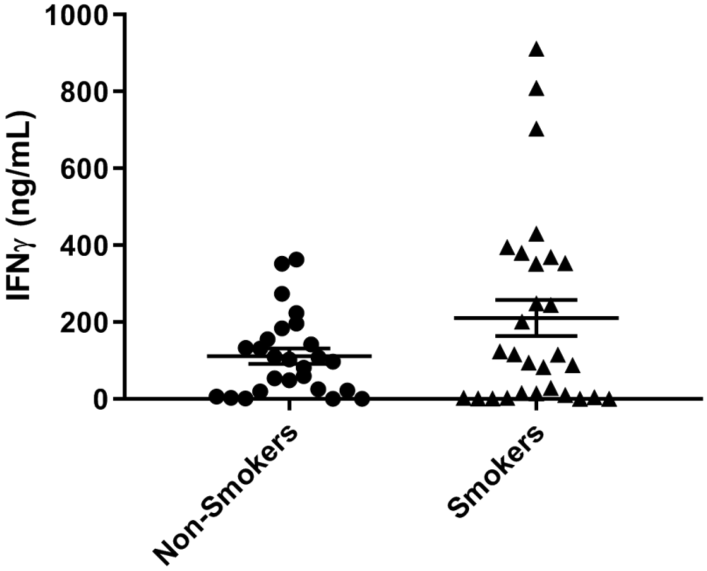

Supplemental Figure S2. Anti-CD3 + anti-CD28 – stimulated PBMCs from smokers tend to generate more IFNγ than non-smokers. Isolated PBMCs were cultured in 10% autologous plasma to simulate *in vivo* conditions for 4 days.

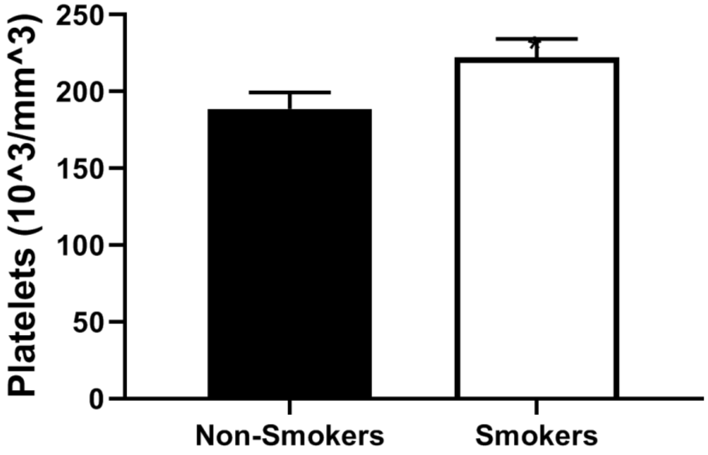

Supplemental Figure S3. Platelet numbers tend to be higher in smokers than non-smokers.

Supplemental Table S1. P values from Pearson correlation analysis between baseline parameters.*

	CRP	Fibrinogen	CEA	WBC	Lymphocyte	Monocyte	Granulocyte	Treg	CD56 NK	Hgb	Hct	RDW	Pct	IL6	IL1β	IL1RA
**CRP**																
**Fibrinogen**	0.757															
**CEA**	0.000	0.635														
**WBC**	0.013	0.002	0.001													
**Lymphocyte**	0.340	0.000	0.126	0.000												
**Monocyte**	0.027	0.002	0.002	0.000	0.000											
**Granulocyte**	0.009	0.027	0.001	0.000	0.001	0.000										
**Treg**	0.609	0.056	0.988	0.038	0.027	0.485	0.069									
**CD56 NK**	0.319	0.068	0.304	0.017	0.034	0.013	0.044	0.106								
**Hgb**	0.807	0.043	0.544	0.050	0.223	0.624	0.041	0.250	0.101							
**Hct**	0.922	0.149	0.344	0.224	0.332	0.714	0.207	0.353	0.105	0.000						
**RDW**	0.372	0.154	0.239	0.150	0.180	0.085	0.234	0.032	0.340	0.145	0.150					
**Pct**	0.068	0.001	0.038	0.000	0.000	0.000	0.000	0.056	0.005	0.692	0.962	0.521				
**IL6**	0.006	0.500	0.004	0.036	0.819	0.034	0.010	0.229	0.583	0.899	0.513	0.781	0.358			
**IL1β**	0.581	0.067	0.821	0.704	0.170	0.868	0.988	0.118	0.520	0.224	0.270	0.764	0.123	0.996		
**IL1RA**	0.578	0.537	0.649	0.802	0.277	0.969	0.497	0.303	0.502	0.118	0.148	0.974	0.430	0.136	0.000	
R =	>0.9	0.8-0.9	0.7-0.8	0.6-0.7	0.5-0.6	0.4-0.5	0.3-0.4	0.2-0.3								

Supplemental Table S2. P values from Pearson correlation analysis between parameters after *E. coli* stimulation of whole blood.*

	CRP	Fibrinogen	CEA	WBC	Lymphocyte	Monocyte	Granulocyte	Treg	CD56 NK	Hgb	Hct	RDW	Pct	TNF-α	IL1β	IL-10	IL-6	MIP-1α
**CRP**																		
**Fibrinogen**	0.757																	
**CEA**	0.000	0.635																
**WBC**	0.013	0.002	0.001															
**Lymphocyte**	0.340	0.000	0.126	0.000														
**Monocyte**	0.027	0.002	0.002	0.000	0.000													
**Granulocyte**	0.009	0.027	0.001	0.000	0.001	0.000												
**Treg**	0.609	0.056	0.988	0.038	0.027	0.485	0.069											
**CD56 NK**	0.319	0.068	0.304	0.017	0.034	0.013	0.044	0.106										
**Hgb**	0.807	0.043	0.544	0.050	0.223	0.624	0.041	0.250	0.101									
**Hct**	0.922	0.149	0.344	0.224	0.332	0.714	0.207	0.353	0.105	0.000								
**RDW**	0.372	0.154	0.239	0.150	0.180	0.085	0.234	0.032	0.340	0.145	0.150							
**Pct**	0.068	0.001	0.038	0.000	0.000	0.000	0.000	0.056	0.005	0.692	0.962	0.521						
**TNF-α**	0.333	0.202	0.262	0.015	0.020	0.037	0.041	0.064	0.238	0.906	0.563	0.534	0.152					
**IL1β**	0.213	0.400	0.142	0.001	0.108	0.039	0.001	0.230	0.063	0.214	0.386	0.253	0.256	0.000				
**IL-10**	0.418	0.806	0.372	0.725	0.418	0.984	0.410	0.616	0.002	0.153	0.242	0.436	0.655	0.371	0.125			
**IL-6**	0.083	0.391	0.065	0.001	0.310	0.055	0.000	0.195	0.012	0.490	0.737	0.224	0.154	0.000	0.000	0.000		
**MIP-1α**	0.084	0.896	0.064	0.006	0.241	0.016	0.003	0.286	0.038	0.926	0.909	0.343	0.228	0.000	0.000	0.006	0.000	
R =	>0.9	0.8-0.9	0.7-0.8	0.6-0.7	0.5-0.6	0.4-0.5	0.3-0.4	0.2-0.3										

Supplemental Table S3. P values from Pearson correlation analysis between parameters after *HSV-1* stimulation of whole blood.*

	CRP	Fibrinogen	CEA	WBC	Lymphocyte	Monocyte	Granulocyte	Treg	CD56 NK	Hgb	Hct	RDW	Pct	TNF-α	IL1β	IL-8	IL-1RA	G-CSF	VEGF	IFN-γ	IL-12p70
**CRP**																					
**Fibrinogen**	0.757																				
**CEA**	0.000	0.635																			
**WBC**	0.013	0.002	0.001																		
**Lymphocyte**	0.340	0.000	0.126	0.000																	
**Monocyte**	0.027	0.002	0.002	0.000	0.000																
**Granulocyte**	0.009	0.027	0.001	0.000	0.001	0.000															
**Treg**	0.609	0.056	0.988	0.038	0.027	0.485	0.069														
**CD56 NK**	0.319	0.068	0.304	0.017	0.034	0.013	0.044	0.106													
**Hgb**	0.807	0.043	0.544	0.050	0.223	0.624	0.041	0.250	0.101												
**Hct**	0.922	0.149	0.344	0.224	0.332	0.714	0.207	0.353	0.105	0.000											
**RDW**	0.372	0.154	0.239	0.150	0.180	0.085	0.234	0.032	0.340	0.145	0.150										
**Pct**	0.068	0.001	0.038	0.000	0.000	0.000	0.000	0.056	0.005	0.692	0.962	0.521									
**TNF-α**	0.134	0.032	0.719	0.216	0.800	0.590	0.141	0.544	0.110	0.002	0.010	0.637	0.574								
**IL1β**	0.175	0.008	0.625	0.184	0.323	0.194	0.217	0.397	0.393	0.059	0.184	0.777	0.546	0.000							
**IL-8**	0.012	0.068	0.000	0.000	0.010	0.003	0.000	0.051	0.074	0.124	0.327	0.704	0.005	0.000	0.001						
**IL-1RA**	0.007	0.073	0.000	0.000	0.026	0.000	0.000	0.332	0.493	0.352	0.783	0.806	0.045	0.051	0.033	0.000					
**G-CSF**	0.176	0.017	0.104	0.022	0.057	0.078	0.037	0.085	0.328	0.014	0.043	0.649	0.418	0.000	0.026	0.000	0.003				
**VEGF**	0.028	0.007	0.001	0.000	0.064	0.000	0.000	0.119	0.043	0.001	0.025	0.135	0.011	0.000	0.000	0.000	0.000	0.006			
**IFN-γ**	0.329	0.006	0.201	0.003	0.173	0.003	0.003	0.203	0.086	0.019	0.096	0.113	0.153	0.000	0.000	0.000	0.001	0.002	0.000		
**IL-12p70**	0.019	0.001	0.001	0.000	0.063	0.000	0.000	0.148	0.015	0.014	0.077	0.048	0.011	0.000	0.000	0.000	0.000	0.007	0.000	0.000	
**PGE2**	0.195	0.001	0.056	0.000	0.008	0.000	0.000	0.432	0.051	0.178	0.487	0.405	0.001	0.000	0.000	0.000	0.000	0.065	0.000	0.000	0.000
R =	>0.9	0.8-0.9	0.7-0.8	0.6-0.7	0.5-0.6	0.4-0.5	0.3-0.4	0.2-0.3													
